# mRNA Transfection of Mouse and Human Neural Stem Cell Cultures

**DOI:** 10.1371/journal.pone.0083596

**Published:** 2013-12-26

**Authors:** Samuel McLenachan, Dan Zhang, Ana Belén Alvarez Palomo, Michael J. Edel, Fred K. Chen

**Affiliations:** 1 Centre for Ophthalmology and Visual Science, The University of Western Australia, Crawley, Western Australia, Australia; 2 Ocular Tissue Engineering Laboratory, Lions Eye Institute, Nedlands, Western Australia, Australia; 3 Control of Pluripotency Laboratory, Molecular Genetics Research Group, Department of Physiological Sciences I, University of Barcelona, Barcelona, Spain; 4 University of Sydney Medical School, Faculty of Medicine, Westmead Childrens Hospital, Division of Pediatrics and Child Health, Sydney, Australia; 5 Ophthalmology Department, Royal Perth Hospital, Wellington Street, Perth, Western Australia, Australia; National University of Singapore, Singapore

## Abstract

The use of synthetic mRNA as an alternative gene delivery vector to traditional DNA-based constructs provides an effective method for inducing transient gene expression in cell cultures without genetic modification. Delivery of mRNA has been proposed as a safer alternative to viral vectors in the induction of pluripotent cells for regenerative therapies. Although mRNA transfection of fibroblasts, dendritic and embryonic stem cells has been described, mRNA delivery to neurosphere cultures has not been previously reported. Here we sought to establish an efficient method for delivering mRNA to primary neurosphere cultures. Neurospheres derived from the subventricular zone of adult mice or from human embryonic stem cells were transfected with EGFP mRNA by lipofection and electroporation. Transfection efficiency and expression levels were monitored by flow cytometry. Cell survival following transfection was examined using live cell counting and the MTT assay. Both lipofection and electroporation provided high efficiency transfection of neurospheres. In comparison with lipofection, electroporation resulted in increased transfection efficiencies, but lower expression per cell and shorter durations of expression. Additional rounds of lipofection renewed EGFP expression in neurospheres, suggesting this method may be suitable for reprogramming applications. In summary, we have developed a protocol for achieving high efficiency transfection rates in mouse and human neurosphere cell culture that can be applied for future studies of gene function studies in neural stem cells, such as defining efficient differentiation protocols for glial and neuronal linages.

## Introduction

The mammalian nervous system contains populations of multipotent stem cells that support ongoing requirements for neurogenesis in the adult brain [1]. Adult neural stem cells can be cultured *in vitro* as floating cell clusters known as neurospheres [2]–[4]. In situ, NSCs supply progenitors that differentiate into specific neuronal subtypes, depending on their anatomical location. However, neurosphere cultures have been shown to display a high degree of plasticity in their differentiation potential [5]–[8]. In addition to the subventricular zone of the brain, human neurosphere cultures can be established from more accessible neural tissues, such as the olfactory epithelium [9], or from mesenchymal and neural crest-derived stem cells in other tissues, including muscle [10], adipose [11], bone marrow [12] and the ocular limbus [13].

In recent years, cellular reprogramming techniques have been developed for the induction of pluripotency in human primary fibroblast cultures through the forced expression of a cocktail of transcription factors, including OCT4, SOX2, C-MYC and KLF4 [14]. Since neurosphere cultures express SOX2 and C-MYC, they may require fewer reprogramming factors [15], or be induced to pluripotency through culture methods alone [16], [17]. These features make neurospheres an attractive candidate as a donor cell for regenerative applications such as cellular reprogramming and tissue engineering.

Cellular reprogramming methods rely on the ability to induce and sustain ectopic gene expression in the donor cells, either through the use of virus-mediated gene transfer [18], [19] or non-viral transfection of DNA vectors [20]. Viral gene delivery methods are undesirable if reprogramming techniques are to be used for clinical applications and transfected DNA can cause mutations through random genomic integration or continue to express genes after reprogramming is completed. For clinical applications mRNA transfection has been proposed as an alternative to DNA-based vectors for inducing gene expression in cell cultures [21]. The mRNA vector provides a method of inducing gene expression without lasting genetic modification of the cell, making it the safest choice for clinical reprogramming applications.

Efficient methods have been described for the delivery of mRNA to human fibroblast cultures [22] as well as dendritic cells [23] and embryonic stem cells [24]. However there are currently no published methods describing the transfection of neurosphere cultures. Here we demonstrate high efficiency mRNA delivery to neurosphere cultures by lipofection and electroporation.

## Materials and Methods

### Ethics Statement

C57BL/6 mice used in this study were bred at the Animal Resources Centre (Murdoch University, WA) and maintained on a 12-hour day/night cycles, with free access to food and water. Principles of laboratory animal care (NIH publication no. 85-23) were followed at all times. All procedures conformed to the Association for Research in Vision and Ophthalmology Statement for the Use of Animals in Ophthalmic and Vision Research and were approved by The University of Western Australia Animal Ethics Committees (Permit No. RA/3/100/853).

### Neurosphere Culture

Tissue was dissected from the subventricular zone of adult C57BL/6 mouse brains as previously described [25]. For routine passaging and culture, neurospheres were grown using the StemPro Neural Stem Cell Serum-Free Media Kit according to the manufacturer’s recommendations (Life Technologies, Carlsbad, CA, USA).

### mRNA Synthesis

**Preparation of template DNA.** Template DNA for *in vitro* transcription was constructed by two-stage PCR and molecular cloning. In the first stage an EGFP DNA fragment was amplified by PCR from plasmid pEGFP-N1 (Clontech, Mountain View, CA, USA) using the following primers: EGFP-fwd: 5′- TAATACGACTCACTATA**G**G**ATG**GTGAGCAAGGGCGAGGAGC; EGFP-rev: 5′- GCCTCCCTCGCGTTATCAGAgATCTAgAgTCgCggCCgCT**TTA**C. First stage PCR produced a product containing a T7 promoter at the 5′ end, and an adaptor sequence at the 3′ end for addition of a polyA-tail. In the second PCR stage, a polyT-tail was added to the 3′end of the EGFP cDNA using the EGFP-fwd primer the PolyT-rev primer (5′-GCCTCCCTCGCGTTATCAGA-(T)^130^). After purification using the PCR Purification Kit (Promega, Fitchburg, WI, USA), the PCR product was cloned into pGEM-T Easy vector (Promega). The resulting pGEMT-EGFP plasmid was transformed into JM109 competent E. coli cells (Promega) and purified using the Qiagen Plasmid Maxi Kit (Qiagen, Venlo, The Netherlands)**.** To prepare template DNA for *in vitro* transcription (IVT), pGEMT-EGFP DNA was linearized using ZraI (Promega) and purified by PCR Cleanup Column (Promega). **In vitro transcription.** Capped single stranded RNA was synthesized via IVT using the MEGAscript-T7 kit (Life Technologies). Generally, 0.8 µg–1µg of purified linear DNA was used in a 20 µl reaction. To obtain a high proportion of capped transcripts, a 4∶1 ratio of 3'-O-Me-m7G(5')ppp(5')G RNA Cap Structure Analog (New England BioLabs, MA, USA):GTP was used in RNA IVT reactions. Reactions were incubated 3-4 hours at 37°C then treated with 1 µl TURBO DNase (Life Technologies) for a further 15-minutes at 37°C before purification on RNeasy MiniSpin columns (Qiagen). RNA products were eluted with 85 µl of H_2_O in each column. To remove 5′ triphosphate moieties from uncapped transcripts, 10 µl of Antarctic Phosphatase reaction buffer and 5 µl (25U) of Antarctic Phosphatase (New England BioLabs) was added to each RNA product and incubated for 30-minutes at 37°C. Synthesized RNA products were then repurified using RNeasy Mini Columns (Qiagen) and quantitated by spectrophotometry and denaturing gel electrophoresis. RNA products were examined by electrophoresis on 2% agarose gels.

### Lipofection

For lipofection experiments, neurospheres were transfected with either Lipofectamine-2000 (LF2000, Life Technologies) or TransIT (Mirus Bio LLC, WI, USA). For each reagent, mRNA:lipid ratios were optimized according to the manufacturers recommendations. For both LF2000 and TransIT, an mRNA:lipid ratio of 1∶3 was found to be optimal (data not shown). For routine lipofection, whole neurospheres were collected from culture flasks 3–4 days after passaging. A sample of the neurosphere suspension was trypisinzed and cell counts performed to determine the cell concentrations. Whole neurospheres were plated at densities of 30 000 cells/well and 100 000 cells/well in 96 and 24 well plates, respectively, and lipoplexes were added on the day of plating. For transfection of adherent cultures, neurospheres were dissociated with TrypLE-Express (Life Technologies) and plated on geltrex (Life Technologies) coated plates at a density of 150,000 cells/cm^2^. EGFP expression in transfected neurospheres was examined using the Olympus IX70 inverted fluorescence microscope and imaged using Olympus DP-Controller 3.1.1.267 acquisition software (Olympus Corporation, Tokyo, Japan). For quantitation of transfection efficiency and fluorescence per cell, neurospheres were trypsinized and analyzed on the FACSCalibur flow cytometer (Becton-Dickinson Biosciences, Franklin Lakes, NJ, USA) as previously described [24]. Flow cytometry data was analyzed using the WinMDI software (Joseph Trotter).

### NEON Electroporation

For electroporation experiments, neurospheres were transfected using the NEON microporation system (Life Technologies). Neurospheres were dissociated using TrypLE Express (Life Technologies) after 3–4 days of growth and resuspended at high cell density in Electroporation Buffer-T. Electroporations were performed in a 10 µl tip containing 100 000 cells and 500ng-2 µg of mRNA. Immediately following electroporation, neurosphere cells were plated into one well in a 24-well plate. Under these conditions, neurospheres were rapidly formed by reaggregation of single cells. Electroporations were optimized using a range of settings according to the manufacturers recommendations. Optimal electroporation results were achieved using a single 20ms pulse at 1350V. Higher voltages were found to induce high levels of cell death (data not shown).

### MTT Cell Proliferation Assay

Neurosphere growth was measured using the MTT (3- [4,5-dimethylthiazol-2-yl]-2,5 diphenyl tetrazolium bromide) assay (Sigma, St. Louis, MO, USA) as previously described [25]. Neurospheres were dissociated by trituration in PBS, seeded into 96-well plates at a density of 30000 cells/well and cultured overnight to allow sphere formation by cell aggregation. Three days after transfection, 10 µl MTT (Sigma, St. Louis, MI, USA) solution (2.5 mg/ml) was added to each well and plates were incubated at 37°C to allow mitochondrial uptake. After 2 hours, cells were lysed by the addition of an equal volume of acidified isopropanol and the absorbance measured at 590nm using the Beckman-Coulter AD200 spectrophotometer (Beckman-Coulter, Brea CA, USA). Within each experiment, absorbances were averaged across 3–6 replicate wells.

### Differentiation Assay

For differentiation experiments, transfected and control neurospheres were plated onto poly-ornithine/laminin coated chamber slides (Becton Dickinson Biosciences, Franklin Lakes, NJ, USA) in StemPro Neural Stem Cell Serum-Free Media (Life Technologies). After overnight culture, the media was replaced without EGF and FGF-2. Slides were fixed for 15 minutes at 37°C in 4% paraformaldehyde 1, 3 and 5 days after plating. Fixed chamber slides were bathed in cold (–20°C) methanol for 15 min at room temperature then washed with PBS. Blocking solution (PBS + 5% normal goat serum + 0.3% Triton) was added and slides incubated at room temperature for 45 min. Primary antibodies were diluted to working concentrations in blocking solution, added to slide chambers and incubated at room temperature for 1 hour. The slides were then washed three times in PBS before the incubation with secondary antibodies and the nuclear marker 4,6-diamidino-2-phenylindole (DAPI). After 1-hour at room temperature, slides were washed and mounted in fluorescent mounting media (Dako, Glostrup, Denmark). Antibodies (and working concentrations) used in these studies were as follows: rabbit anti-Ki67 (1∶500, Abcam), mouse anti-βIII-Tubulin (1∶500, Promega), rabbit anti-Nestin (1∶250, Abcam), rabbit anti-GFAP (1∶500, Dako), goat anti-mouse IgG-AlexaFluor594 (1∶500, Life Technologies) goat anti-rabbit-IgG-AlexaFluor488 (1∶500, Life Technologies) and goat anti-rabbit-IgG-AlexaFluor594 (1∶500, Life Technologies).

### RT-PCR Analysis

Total RNA was extracted from neurospheres using the AllPrep RNA/Protein Kit (Qiagen). Total RNA was then quantified using the Nanodrop 2000 spectrophotometer (ThermoFisher Scientific, Waltham, MA, USA). First-strand cDNA was synthesized using 1–3 µg of RNA and SuperScript III Reverse Transcriptase (Life Technologies) according to the supplier's protocol. RT-PCR reactions for mouse IFNβ and GAPDH were performed in a 10 µl mixture containing 1 µl (50–100 ng) of cDNA, primers (10 µM) and Rotor-Gene SYBR-Green RT-PCR Master Mix (Qiagen) in the 9600 GeneAmp PCR thermocycler (Perkin Elmer, Waltham, MA, USA). RT-PCR reactions were examined by gel electrophoresis on 1% agarose. Primer sequences used were: Mouse IFNβ: Fwd 5′-GGAGATGACGGAGAAGATGC, Rev 5′-CCCAGTGCTGGAGAAATTGT; Mouse GAPDH: Fwd 5′-GGTGAAGGTCGGTGTGAACG, Rev 5′-CTCGCTCCTGGAAGATGGTG.

### Cell Cycle Analysis

Cell cycle analysis was performed as previously described [26]. Neurospheres were harvested and dissociated into single cells by brief incubation in TrypLE Express (Life Technologies). Cells were then added dropwise into another tube containing ice-cold 100% ethanol mixing on a vortex, and incubated on ice for 10 minutes. Ethanol was then removed and cells were resuspended in ice-cold phosphate buffered saline (PBS) containing 2% fetal calf serum, RNase1 (100 µg/ml), and propidium iodide (PI, 50 µg/ml). DNA analysis was then performed using FACSCalibur flow cytometer (Becton-Dickinson Biosciences).

## Results and Discussion

**Figure 1:**
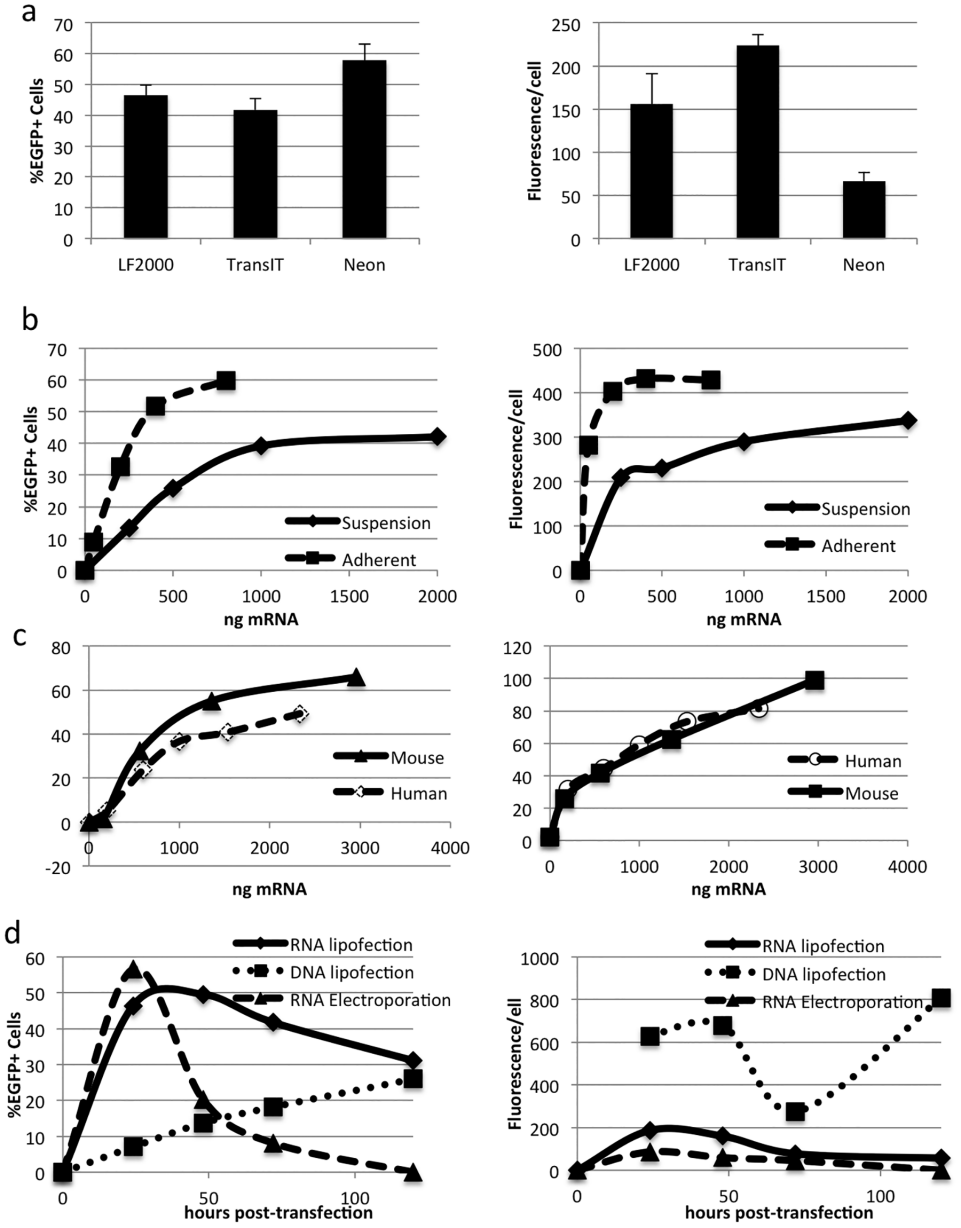
Transfection of Mouse Neurospheres with EGFP mRNA. **A:** Adult mouse neurospheres were transfected in 24 well plates with 500ng of EGFP mRNA using the lipofection reagents Lipofectamine 2000 or Transit or with 1 µg of EGFP mRNA using the NEON microporation system. Transfection efficiency (left panel) and EGFP fluorescence per cell (right panel) were measured by flow cytometry. 24 hours after transfection. **B:** Mouse neurospheres were cultured as either adherent monolayers (dashed line) or floating neurospheres (solid line) in 24 well plates and transfected with increasing amounts of EGFP mRNA lipoplexes. Transfection efficiency (left panel) and EGFP fluorescence per cell (right panel) were measured by flow cytometry. **C:** Whole mouse (solid line) or human (dashed line) neurospheres were transfected with increasing concentrations of mRNA using the NEON microporation system and seeded into 24 well plates. Transfection efficiency (%EGFP, dashed line) and EGFP fluorescence per cell (MPF, solid line) were measured by flow cytometry. **D:** Mouse neurospheres were transfected with 500ng of EGFP mRNA lipoplexes (solid line), pEGFP-N1 DNA lipoplexes (dotted line) or 1 µg of EGFP mRNA using the NEON microporator (dashed line). Transfection efficiency (left panel) and EGFP fluorescence per cell (right panel) were measured by flow cytometry 24, 48, 72 and 120 hours after transfection.

To select a method for mRNA delivery, mouse neurospheres were transfected with EGFP-mRNA prepared by *in vitro* transcription using a lipofection system (LipofectAMINE-2000 (LF2000) or TransIT), or the NEON microporation system. EGFP expression was measured by flow cytometry after 24-hours. Lipofection of neurospheres typically yielded transfection efficiencies of up to 40–50%. In comparison, electroporation yielded higher transfection efficiencies (60–70%), with lower EGFP expression per cell (Figure 1a). Increasing the concentration of mRNA lipoplexes during lipofection or free mRNA during microporation did not increase transfection efficiency, but did result in small increases in EGFP fluorescence per cell (Figure 1b-c). Lipofection efficiencies were maximal at 50–100ng mRNA/well in a 96-well plate, and at 500ng-1 µg mRNA/well in a 24-well plate. (Figure 1b and data not shown). Electroporation efficiency was optimal using 1 µg of mRNA per 100000 neurospheres cells (Figure 1c).

Since neurospheres grow as cell clusters in suspension, accessibility of lipoplexes to the cells may become more restricted as neurospheres get larger. Indeed, transfection efficiency was increased in small neurospheres grown for 3 days in culture, compared with large neurospheres grown for 7 days in culture (50% vs 20%, data not shown). To increase lipoplex accessibility to neural progenitor cells, neurospheres were plated onto extracellular matrix and cultured as adherent monolayers. Higher transfection efficiencies were achieved with lower mRNA lipoplex concentrations in neurosphere monolayers (60%) compared with whole neurospheres in suspension (40%). However, transfection efficiency could not be further increased through addition of more lipoplexes in either sphere cultures or adherent monolayers (Figure 1b). While, floating neurospheres tolerated additional lipoplexes, adherent neurosphere monolayers showed signs of significant cell death at mRNA lipoplex concentrations above 800ng/well in a 24-well plate (Figure 1b). Increasing mRNA concentration during electroporation did not increase transfection efficiencies and a population of cells (30–40%) failed to express EGFP (Figure 1c). These results suggest that a population of neurosphere cells do not take up mRNA, as previously reported during mouse embryonic stem cell transfection [24].

Following a single lipofection of EGFP mRNA, bright green fluorescence was detected in approximately 50% of transfected mouse neurosphere cells for around 48-hours, after which both the number of EGFP expressing cells and the intensity of the signal diminished (Figure 1d).

In comparison, lipofection of neurospheres with the pEGFP-N1 plasmid was less efficient than EGFP mRNA. Following DNA transfection, transfection efficiencies increased over the 5-day time course examined (Figure 1d). Despite lower transfection efficiencies, plasmid DNA yielded brighter EGFP fluorescence than mRNA in transfected cells, suggesting strong activity of the CMV-IE promoter in mouse neurosphere cells. 3-days after DNA transfection EGFP expression per cell decreased by half, while transfection efficiency continued to increase during this time, suggesting the division of transfected cells and dilution of both EGFP protein and transfected plasmid. After 5 days, EGFP levels in DNA transfected neurosphere cells had recovered to maximal levels indicating continued expression of EGFP from retained plasmids (Figure 1d).

In contrast to DNA transfection, mRNA lipofection elicited a transient wave of EGFP expression. Both transfection efficiency and EGFP expression per cell reached their peak values at 24–48 hours and dropped markedly at 72-hours (Figure 1d), consistent with the degradation of the EGFP mRNA and protein. Microporation resulted in lower levels of EGFP expression than lipofection and a shorter duration of EGFP expression, with peak transfection efficiencies observed 24-hours after electroporation (Figure 1d).

**Figure 2:**
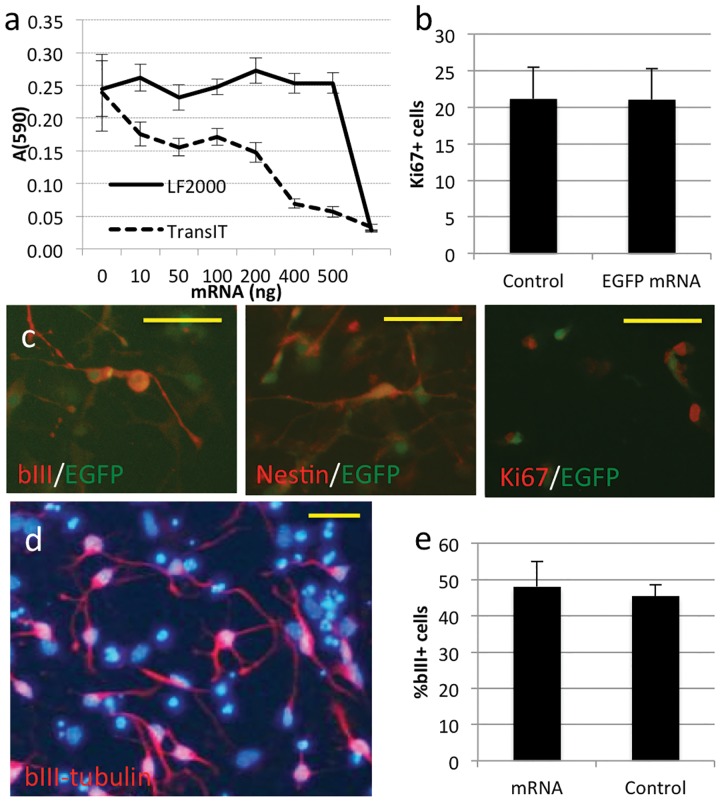
Lipofection of EGFP mRNA in Mouse Neurospheres. **A:** Adult mouse neurospheres were cultured in 96 well plates and transfected with increasing concentrations of mRNA lipoplexes using Lipofectamine 2000 or TransIT. Two days after transfection, cell numbers were quantitated using the MTT assay. **B:** Mouse neurospheres were plated onto geltrex-coated 24 well plates 24 hours after transfection with 500ng of mRNA lipoplexes. After overnight culture, resulting monolayers were fixed and the proportion of proliferating cells determined by Ki67 immunostaining. Each bar represents the mean percentage of Ki67^+^ cells from 6-8 transfections. Error bars indicate standard deviation. **C:** Transfected mouse neurospheres were plated as described in B and immunostained for Ki67, Nestin or βIII-Tubulin. Micrographs show merged images consisting of the antibody signal (red), EGFP fluorescence (green). Scale bars indicate 50 µm. **D:** Mouse neurospheres were transfected with 500ng of mRNA lipoplexes, plated onto geltrex and cultured without mitogens for 3 days before immunostaining for βIII-Tubulin (red) and DAPI (blue). Scale bar indicates 50 µm. **E:** Quantitation of neuronal cells. Each bar represents the mean percentages of βIII-Tubulin^+^ cells from 3 transfections. Error bars indicate standard deviation.

LF2000 lipoplexes were well tolerated by mouse neurospheres over a range of concentrations. 24-hours after lipofection, neurospheres transfected with increasing concentrations of LF2000-mRNA lipoplexes had similar numbers of viable cells as untransfected controls. LF2000 lipoplexes were toxic only at very high concentrations (600ng/well in a 96-well plate). In contrast, mouse neurospheres displayed dose dependent toxicity when exposed to lipoplexes formed using the TransIT reagent (Figure 2a).

To determine whether lipofection of mRNA affected proliferation or differentiation potential, mouse neurospheres were transfected with EGFP mRNA before plating onto geltrex coated chamber slides for monolayer culture. 24-hours after plating, neurosphere monolayers were fixed and immunostained for the neural progenitor marker Nestin or the proliferation marker Ki67. Nestin immunolabelling was observed in the majority of cells and was unaffected by EGFP-mRNA lipofection (data not shown). Similar proportions of cells (20%) were immunopositive for Ki67 in transfected and control monolayers, indicating that lipofection of EGFP-mRNA did not induce cell cycle exit (Figure 2b). EGFP fluorescence was present in cells that were immunopositive for Nestin, Ki67, βIII-tubulin (Figure 2c) or GFAP, suggesting lipoplex uptake was not limited to or excluded by different populations of progenitors within the neurosphere.

To induce differentiation, EGF and FGF2 were removed from the culture media 24-hours after plating. 3-days after growth factor removal, transfected neurospheres yielded similar proportions of βIII-tubulin expressing neurons as untransfected controls (Figure 2d-e). After five days, expression of GFAP in differentiating cultures was also unaltered by transfection (data not shown). Together, the above data demonstrate that LF2000 provides a high efficiency, low toxicity method for mRNA lipofection of neurosphere cultures that does not alter the proliferation or differentiation of the transfected neural progenitors.

For cellular reprogramming applications, the persistence of artificial DNA constructs in reprogrammed cells is disadvantageous for a number of reasons, including incomplete silencing or insertional mutagenesis of transgenes. The degradation of transfected mRNA by endogenous pathways provides an elegant solution to these issues, enabling the induction of exogenous gene expression without genetic modification of the cell. However, the transience of the mRNA signal after lipofection also represents a technical challenge for reprogramming applications, which require sustained expression of transcription factors over longer periods of time than can be achieved by a single lipofection. To overcome this problem, mRNA reprogramming protocols incorporate multiple transfections.

**Figure 3:**
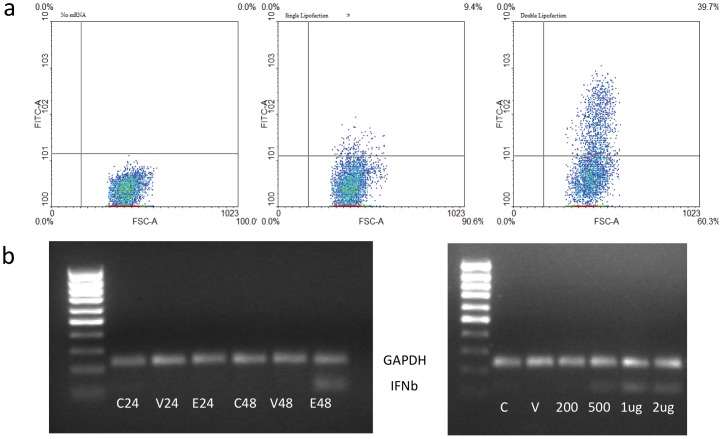
Multiple Transfection of Mouse Neurospheres. **A:** Adult mouse neurospheres were cultured as whole NS in 24 well plates and transfected with 500ng of EGFP mRNA lipoplexes. Two days after the first transfection, a second dose of lipoplexes was applied to the cultures. Transfection efficiencies were measured by flow cytometry and fluorescence microscopy 72(left panel), a single dose (middle panel) or two doses (right panel) of mRNA lipoplexes. **B:** Total RNA was extracted adult mouse neurospheres and IFNβ expression measured by RT-PCR (lower bands) 24 hours after transfection. RT-PCR for GAPDH mRNA (upper band) was performed as a positive control. Lanes in the left panel show untransfected control neurospheres (C-), neurospheres treated with LF2000 alone (V-) or with EGFP mRNA lipoplexes (E-) after a single (24) or double (48) dose of lipoplexes. The right panel shows IFNβ expression after a single transfection of increasing amounts (0, 200ng, 500ng, 1 µg, 2 µg) of EGFP mRNA lipoplexes. The 1 kb Plus DNA Ladder (Life Technologies) is shown in the first lane of each panel.

During suspension culture neurospheres are formed from single cells over 3–7 days before reaching sizes of 200–400 µm and requiring further passaging. Taking the growth and expression kinetics into account, we opted for a double transfection protocol to sustain expression over 4–5 days of neurosphere growth. Small neurospheres (<100 µm) were transfected with EGFP-mRNA 3-days after passaging, resulting in EGFP expression in 40–60% of cells. As shown above, EGFP expression in single transfected wells had declined markedly 72-hours after lipofection, however, the application of a second dose of mRNA lipoplexes at 48-hours restored the population of green fluorescent cells observed by flow cytometry at 72-hours, indicating fresh uptake of mRNA lipoplexes (Figure 3a).

Transfected mRNA can activate intracellular RNA-receptors, inducing signal transduction and expression of type 1-interferon (IFN) genes. In fibroblasts, activation of IFN receptors after mRNA transfection primes cells for apoptosis on subsequent transfection, limiting the efficacy of repeated transfection protocols [27], [28]. Using our multiple lipofection protocol, we observed the induction of IFNβ expression by RT-PCR in double transfected, but not single transfected neurospheres (Figure 3b, left panel). However, increasing the lipoplex concentration during a single transfection resulted in a dose dependent increase in IFNβ expression. Low lipoplex concentrations (200 ng/well) resulted in no IFNβ mRNA detected, while concentrations above 500 ng/well induced robust IFNβ expression (Figure 3b, right panel). In mouse neurospheres, IFNβ has been shown to activate JAK-STAT signaling, increase expression of the Cdk inhibitor p27 and inhibit proliferation [26]. Therefore, additional experiments are warranted to determine whether the use of modified mRNA [28] or siRNA targeting molecules involved in IFN signaling [27] can be applied to neurospheres to prevent IFNβ induction.

**Figure 4:**
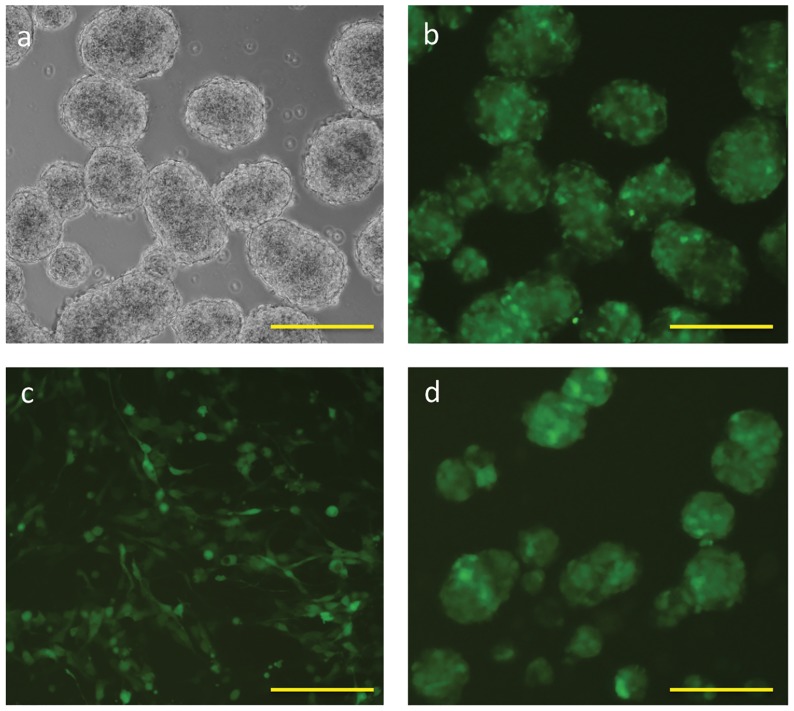
mRNA Transfection of Human Neurospheres. **A-D:** Human embryonic stem cell-derived neurospheres were transfected using LF2000 in suspension culture (**A-B**) or adherent monolayers (**C**) or using NEON electroporation (**D**) Micrographs were taken 24 hours after transfection. Scale bars indicate 250 µm. **E:** Human neurospheres were transfected with 500 ng of EGFP mRNA lipoplexes in 24 well plates. Two days after the first transfection, a second dose of lipoplexes was applied to the cultures. Transfection efficiencies were measured by flow cytometry and fluorescence microscopy 72 hours after the first transfection. Flow cytometry plots show neurospheres receiving no lipoplexes (left panel), a single dose (middle panel) or two doses (right panel) of mRNA lipoplexes. Insets in **E** show fluorescence micrographs of human neurospheres.

In parallel experiments, human embryonic stem cell-derived neurospheres (hENS) performed similarly to mouse neurospheres when transfected with EGFP mRNA by lipofection or microporation (Figure 1c, 4a-d). In multiple transfection experiments, a second application of mRNA lipoplexes 48-hours after transfection could also be used to sustain EGFP expression in transfected hENS neurospheres (Figure 4e).

**Figure 5:**
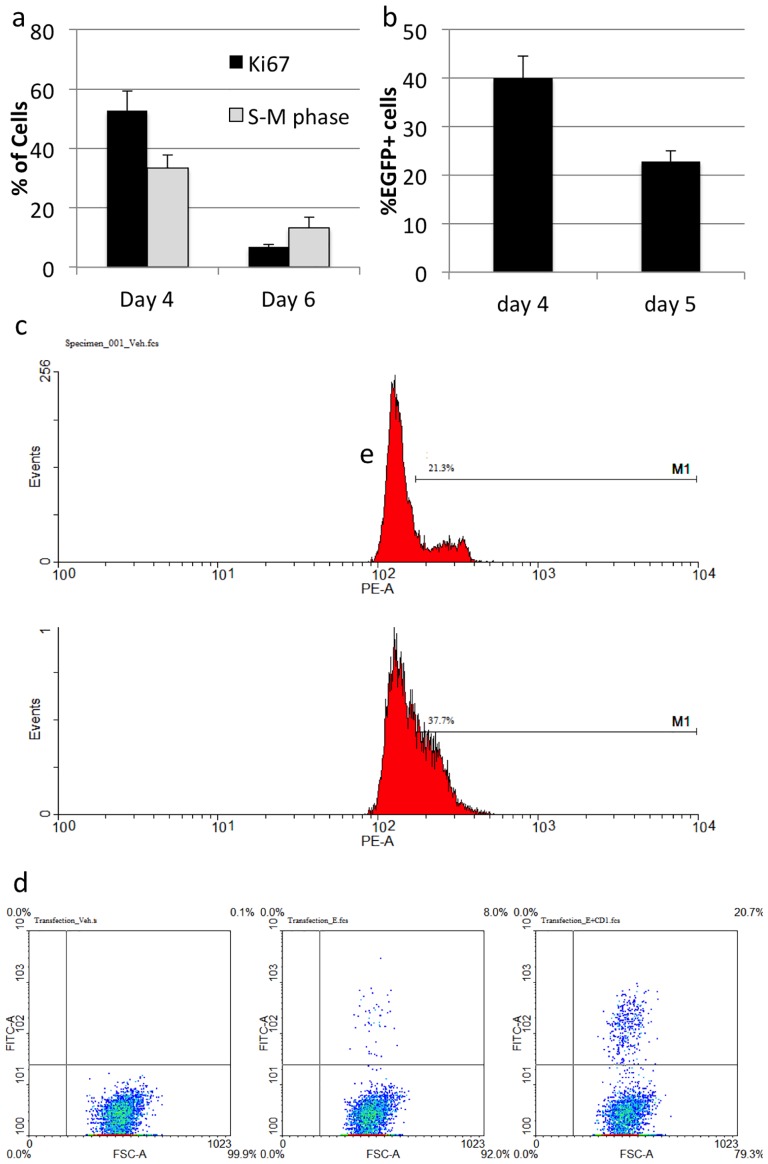
Cyclin D1 enhances mouse neurosphere transfection. **A:** Adult mouse neurospheres were seeded as single cells and cultured for 3 or 6 days. Day 4 neurospheres and day 6 neurospheres were dissociated, fixed and stained with PI for cell cycle analysis by flow cytometry (black bars) or were plated onto geltrex and stained for Ki67 (white bars). **B:** Mouse neurospheres were transfected with 500ng EGFP mRNA on day 4 or day 5 after seeding as single cells. Transfection efficiencies were measured by flow cytometry after 24 hours. **C:** Day 3 adult mouse neurospheres were transfected with 500ng of CyclinD1 or mRNA lipoplexes (lower plot) or LF2000 alone (upper plot). 24 hours after transfection, cells were dissociated and cell cycle profiles obtained by PI staining and flow cytometry. **D:** Day 6 mouse neurospheres were transfected with 1 µg of mRNA lipoplexes containing either EGFP mRNA or EGFP mRNA plus Cyclin D1 mRNA (500ng each). Transfection efficiency was measured by flow cytometry 24 hours later.

In culture, neurospheres are formed from single cells that grow into clusters of dividing progenitors and small populations of stem cells. The initial logarithmic growth phase lasts for 4 days, with neurospheres reaching maximum sizes within 5-6 days. Both Ki67 immunostaining and cell cycle analysis by flow cytometry revealed a large reduction in the number of proliferating cells after 6 days of mouse neurosphere growth compared with 4 days of neurosphere growth (**Figure 5a**). We found mRNA lipofection efficiency was similarly reduced in neurospheres cultured for 5 days compared with neurospheres cultured for 4 days (**Figure 5b**). These results suggest that lipoplex uptake by neurospheres may be dependent on proliferation, as previously observed in other cell types [24].

To enhance proliferation in mouse neurospheres, we transfected cells with Cyclin-D1 mRNA. 24 hours after transfection, neurospheres were dissociated, fixed and stained with PI for cell cycle analysis by flow cytometry. While 21% of cells treated with LF2000 alone were found to be in S-M phase of the cell cycle, transfection with Cyclin D1 mRNA increased the proportion of actively dividing cells to 37% (**Figure 5c**).

To further explore the relationship between lipofection efficiency and neurosphere growth, we transfected mouse neurospheres with 1 µg of EGFP mRNA 6 days after seeding. Transfection efficiency was only 8% in day 6 neurospheres, consistent with the number of Ki67+ cells present at this time (**Figure 5a, d**). However, transfection of day 6 mouse neurospheres with lipoplexes containing 500ng of EGFP mRNA and 500ng of CyclinD1 mRNA increased EGFP transfection efficiency to 20% (**Figure 5d**). Together, these results suggest CyclinD1 mRNA promoted cell division in transfected neural progenitor cells.

The similarity in transfection kinetics between mouse and human neurospheres could suggest a general applicability of these methods to neurosphere cultures of diverse origin. Indeed, neurospheres cultured from the adult human or mouse ocular limbus could also be transfected using the methods described here (data not shown). Since neurospheres may be easily derived from a number of tissues that are accessible in adult human patients [9]–[12], they may represent a useful source of autologous donor cells for cellular reprogramming applications, including the induction of pluripotent stem cell lines. The use of mRNA transfection for inducing gene expression in neurospheres avoids any risks associated with viral or DNA vectors, providing a method suitable for clinical application. Our results demonstrate that multiple doses of mRNA lipoplexes can be used to sustain gene expression during neurosphere growth. Taken together, our findings suggest a transfection regime consisting of 2–3 doses of 500-1 µg mRNA lipoplexes with an mRNA:LF2000 ratio of 1:3 at 48-hour intervals is suitable to sustain EGFP gene expression in mouse and human neurospheres cultures.
